# A Rare Case of Bullous Lichen Planus in a Young Male Patient With Nephrotic Syndrome

**DOI:** 10.7759/cureus.85837

**Published:** 2025-06-12

**Authors:** Sulhera Khan, Sonia Golani, Zara Saeed, Humaira Talat, Nazish Shah

**Affiliations:** 1 Dermatology, Dow University of Health Sciences, Civil Hospital Karachi, Karachi , PAK; 2 Internal Medicine, Jinnah Postgraduate Medical Centre, Karachi, PAK; 3 Dermatology, Dow University of Health Sciences, Civil Hospital Karachi, Karachi, PAK

**Keywords:** bullous lichen planus, childhood nephrotic syndrome, nephrotic syndrome, renal failure, vesiculobullous lesions

## Abstract

Lichen planus (LP) is a chronic, immune-mediated papulosquamous dermatosis that affects the skin, mucous membranes, and nails. Bullous LP (BLP) is a rare morphological variant characterized by the development of vesicles and bullae, either arising de novo or over existing LP lesions. We present a rare and noteworthy case of a young male patient with nephrotic syndrome who developed pruritic and painful bullous lesions. The initial clinical presentation was atypical and led to diagnostic uncertainty, with multiple differential diagnoses considered. However, histopathological evaluation ultimately confirmed the diagnosis of BLP. This case is of particular significance as it represents one of the first documented associations between BLP and underlying nephrotic syndrome with renal failure. The rarity of this presentation highlights the need for a high index of suspicion when evaluating bullous eruptions, especially in patients with systemic comorbidities. Accurate diagnosis through clinicopathological correlation, including histopathology and direct immunofluorescence, is essential for appropriate classification and management. This case contributes to the limited literature on BLP and emphasizes the importance of thorough evaluation in atypical dermatologic presentations.

## Introduction

Lichen planus (LP) is a papulosquamous inflammatory dermatosis mediated by T cells affecting the skin, mucous membranes, and nails [1]. LP is derived from a Greek word, "leichen," which means tree moss, and a Latin word, "planus," which means flat, even [2]. The disease was first described by Dr. Wilson in 1869 [3]. It is characterized by a prevalence of 0.22%-5% [4]. It is commonly seen in middle-aged adults of both genders. Some studies highlight a slight female preponderance [5]. LP is characterized by six P’s: papules, plaques, pruritus, purple, polygonal, and planar [1]. It is also characterized by the presence of Wickham’s striae, which are fine, white-grey lines on the lesions, more commonly described on mucosal lesions [6]. There are various subtypes of LP based on morphology, which include papular (classic), hypertrophic, vesiculobullous, actinic, annular, atrophic, linear, follicular, LP pigmentosus, and LP pigmentosus-inversus [4]. Among these, bullous LP is a rare variant of LP that is characterized by the development of purple vesicles and bullae on the skin and mucosal surfaces and may be associated with pain or burning sensations. It can develop independently or in the setting of preexisting LP lesions. The exact prevalence of the disease is unknown, but it is known to be low among the different variants of LP [7]. It is also rarely reported in children.

We report a case of a young male patient with nephrotic syndrome and severe renal failure who presented with atypical, pruritic, and painful bullae and blisters. Initially misdiagnosed with several conditions that mimic bullous LP (BLP), he was ultimately diagnosed with BLP based on histopathological findings. This case is significant due to the rare presentation of BLP in a young male patient with underlying renal failure.

## Case presentation

A seven-year-old male patient presented to the dermatology outpatient department with a known history of nephrotic syndrome, diagnosed at the age of three years secondary to IgM nephropathy, for which he had previously received steroid therapy. He also had secondary hypertension related to his renal condition, which was currently being managed with a beta-blocker. Additionally, he has a past medical history of cerebral venous sinus thrombosis, which occurred one year ago and was treated with rivaroxaban and low molecular weight heparin.

**Figure 1:**
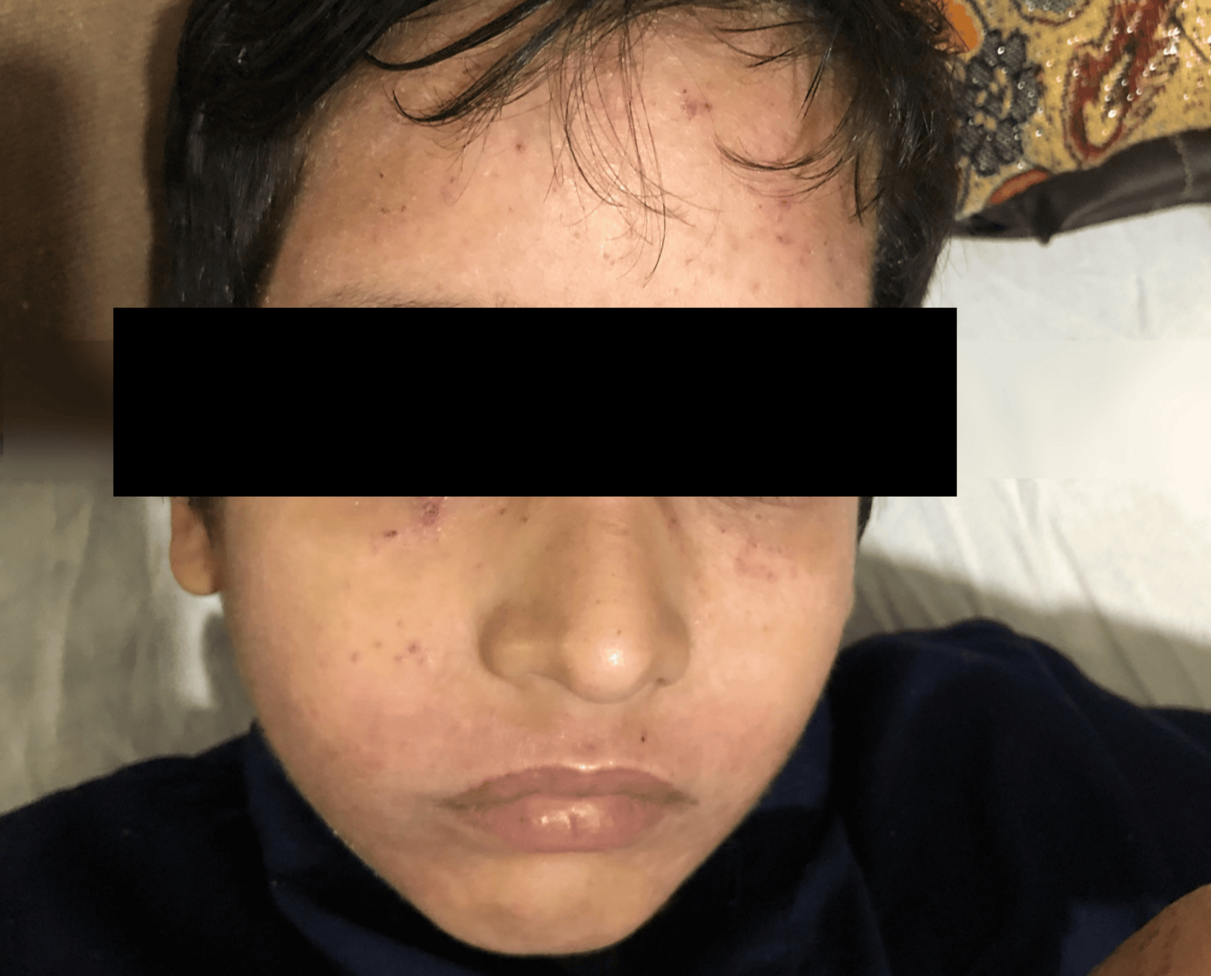
Generalized facial swelling with scattered papules predominantly on the forehead and around the eyes (periorbital region) The parents of the patient consented to have the patient's images used in an open-access publication. A written and signed consent statement from the patient's parents was provided to the journal.

**Figure 2:**
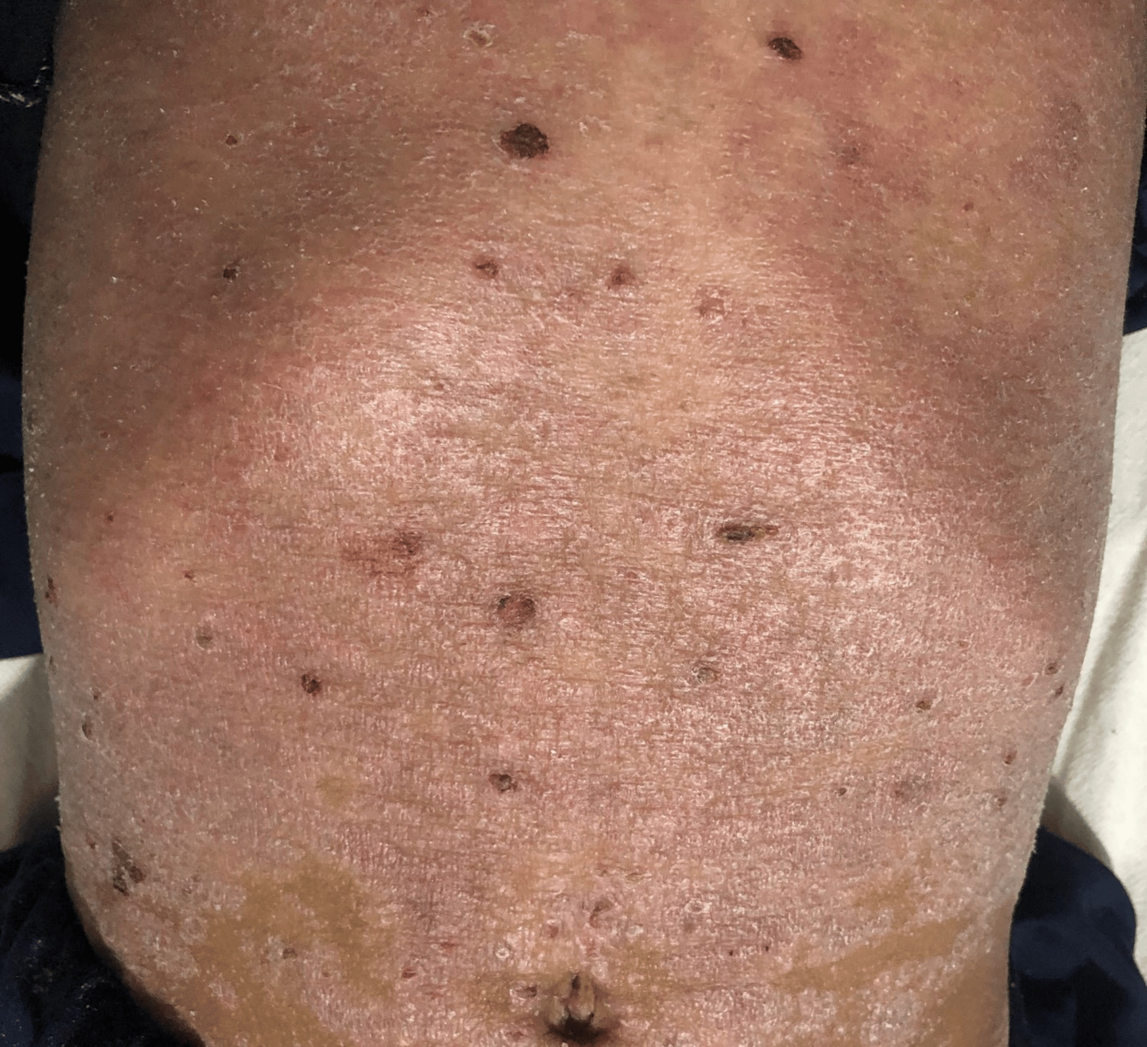
Generalized scaling of the abdomen with scattered hemorrhagic crusts

**Figure 3:**
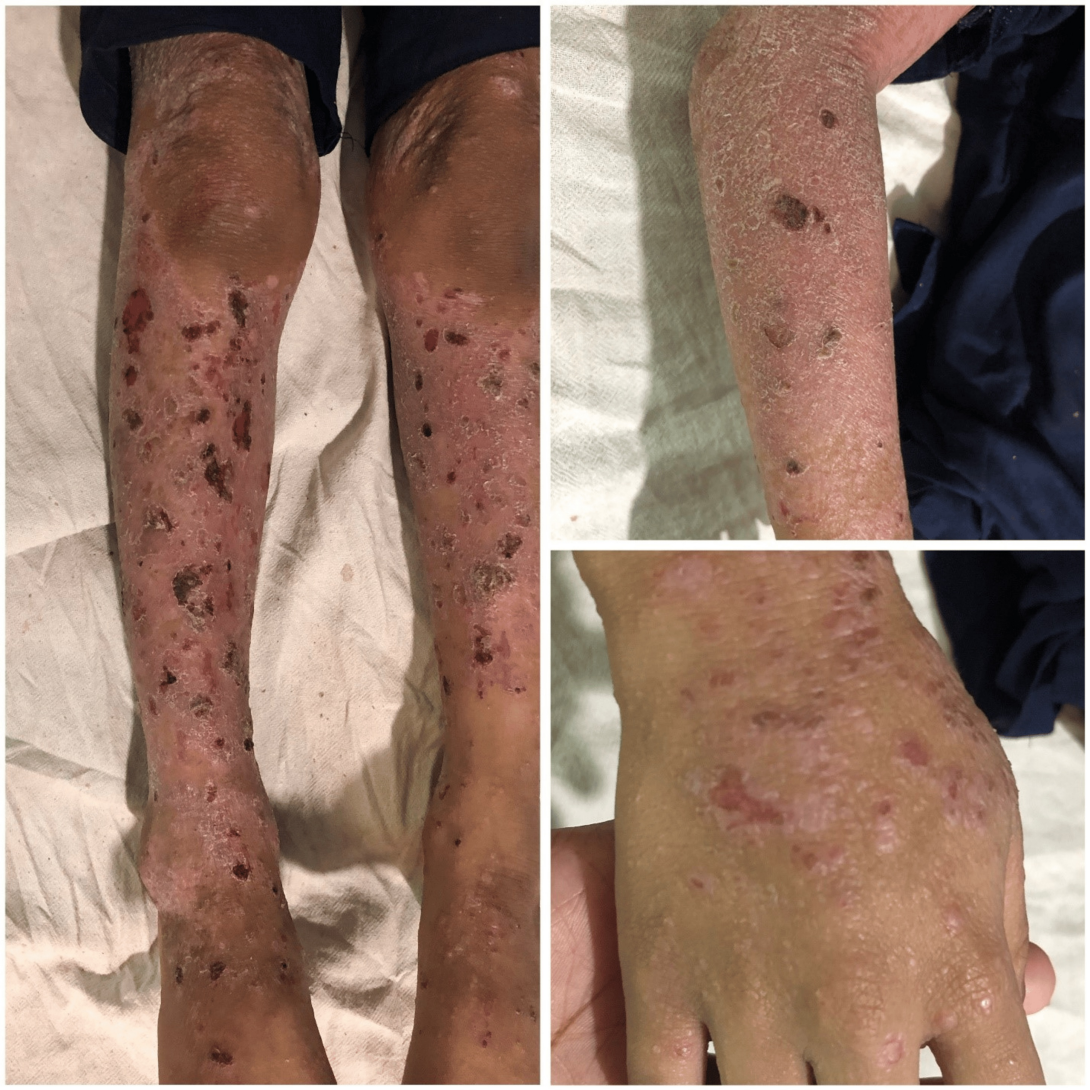
Presence of hemorrhagic crusts, raw erosions, and large scaly plaques on the legs. Close-up examination of the hands reveals generalized scaling, crusted papules on the forearms, and small, tense blisters on the dorsum of the hands.

The patient presented with chief complaints of erythematous, scaly dermatitis affecting the entire body over the past two months. He reported being in his usual state of health until two months ago, when he initially noticed erythematous scaling on his legs accompanied by pruritus. The rash gradually progressed, spreading across the body with the appearance of small erythematous to violaceous papules, some of which coalesced into plaques. The eruption also involved the face and scalp, showing a seborrheic distribution with marked scalp scaling and excoriations. Later, hemorrhagic tense blisters evolved on or near the affected skin, which ruptured spontaneously, leading to the formation of hemorrhagic crusts (Figures 1-3).

Upon further questioning, the patient denied any worsening of lesions with sun exposure. There was no recent use of new prescription, over-the-counter, or herbal medications, and no history of lesion flare-ups following trauma or recent vaccinations. Additionally, there was no documented family history of similar dermatologic conditions. Growth assessment revealed mild growth retardation, with the patient falling below the normal percentile range for both weight and height.

An initial clinical diagnosis considered various differential possibilities, including scabies, acute LP, BLP, eczema, and psoriasis. A laboratory workup was conducted, which revealed an elevated white blood cell (WBC) count of 15 × 10⁹/L (normal range: 5.0 - 13.0 × 10⁹/L), an increased C-reactive protein (CRP) level of 25 mg/L (normal: <10 mg/L), and a mildly raised serum creatinine of 1.1 mg/dL (normal: 0.3 - 0.7 mg/dL). Liver function tests, erythrocyte sedimentation rate (ESR), and a detailed urine report were within normal limits. The laboratory investigations are summarized in Table 1.

**Table 1:** Laboratory investigations of our patient

Parameter	Patient value	Reference range
Hemoglobin	13.0 g/dL	11.5 – 15.5 g/dL
White blood cell count (WBC)	15 × 10⁹/L	5.0 – 13.0 × 10⁹/L
Platelets	280 × 10⁹/L	150 – 450 × 10⁹/L
Alanine transaminase (ALT)	25 U/L	< 45 U/L
Aspartate transaminase (AST)	30 U/L	< 45 U/L
Total bilirubin	0.6 mg/dL	0.1 – 1.0 mg/dL
Direct bilirubin	0.2 mg/dL	0.0 – 0.3 mg/dL
Alkaline phosphatase (ALP)	320 U/L	150 – 420 U/L
Urea	25 mg/dL	10 – 40 mg/dL
Creatinine	1.1 mg/dL	0.3 – 0.7 mg/dL
Sodium	140 mmol/L	135 – 145 mmol/L
Potassium	4.2 mmol/L	3.5 – 5.1 mmol/L
Bicarbonate	24 mmol/L	22 – 28 mmol/L
Chloride	102 mmol/L	98 – 107 mmol/L
Hepatitis B surface antigen (HBsAg)	Negative	
Anti-hepatitis C (HCV)	Negative	
Human immunodeficiency virus (HIV)	Negative	
Erythrocyte sedimentation rate (ESR)	8 mm/hr	<10 mm/hr
C-reactive protein (CRP)	25 mg/L	<10 mg/L
Detailed urine report	Normal	
Pus culture (skin)	Methicillin-resistant *Staphylococcus aureus* (sensitive to linezolid and vancomycin)	

Skin cultures grew methicillin-resistant *Staphylococcus aureus* (MRSA), which was found to be sensitive to vancomycin and linezolid. Given the patient’s pre-existing renal disease and the associated risk of nephrotoxicity with vancomycin, treatment was initiated with intravenous linezolid. Serological testing for hepatitis B, hepatitis C, and HIV was negative. Once the skin infection resolved and repeat cultures were negative, a skin biopsy was performed.

Histopathological examination showed characteristic features including hyperkeratosis, hypergranulosis, and acanthosis with irregular saw-toothed rete ridges. There was prominent liquefactive degeneration of the basal cell layer, resulting in the formation of Max-Joseph spaces and subepidermal blister formation. These findings were highly suggestive of BLP. Direct immunofluorescence testing was negative, further supporting the diagnosis and ruling out other immunobullous disorders.

The patient was treated with potent topical corticosteroids (clobetasol propionate 0.05% and betamethasone valerate 0.1% ointment) applied to the blistered areas. For the raw erosions and crusted lesions, emollients (50% white soft paraffin and 50% liquid paraffin) were used, along with topical fusidic acid to prevent infection. Given the patient’s underlying renal condition, a nephrology consultation was sought, and a collaborative treatment plan was developed. Upon follow-up, there was marked improvement, with resolution of most of the lesions and pruritus and no development of new blisters, leading to a significant enhancement in the patient's quality of life.

## Discussion

BLP is a rare, T-cell-mediated variant of LP, marked by the targeted migration of disease-specific T-cells against basal keratinocyte antigens. This immune response triggers a cytokine cascade and inflammation, leading to a dense CD4+ and CD8+ T-cell infiltrate and subsequent keratinocyte apoptosis [7]. Blister formation occurs because of pronounced liquefaction and degeneration of basal cells, along with a characteristic band-like inflammatory infiltrate in the papillary dermis [8]. BLP is classified into two variants: familial and non-familial. While the condition most commonly occurs sporadically, familial cases tend to present at a younger age and are often associated with nail involvement [7].

While LP typically involves the flexural surfaces, BLP more frequently affects the oral mucosa and lower extremities, with or without nail involvement. Blisters usually develop near the classic LP lesions. Wickham’s striae are commonly observed in these lesions, which often appear bilaterally [9]. The blisters are usually tense and multiloculated [10].

In addition to cutaneous involvement, BLP has been reported as an isolated presentation on the oral mucosa, appearing as hyperkeratotic and erosive plaques with characteristic Wickham’s striae [9]. Nail involvement, including hemorrhagic crusting and nail loss, has also been documented [11]. There is a case report of BLP developing at skin graft donor sites in a patient with psoriasis [12], as well as following hypersensitivity reactions to radiocontrast media used in intravenous pyelography [13]. Associations between BLP and conditions such as systemic sclerosis, scabies, and the hepatitis B vaccine have also been reported [7]. However, to date, there have been no documented cases linking BLP with renal failure, making our case a novel presentation.

BLP can closely mimic several other blistering and inflammatory skin conditions, making accurate diagnosis challenging. One of the primary mimics is LP pemphigoides (LPP), in which the blisters often arise on unaffected skin rather than on preexisting LP lesions and are caused by autoantibodies targeting BP180 (type XVII collagen) at the basement membrane zone. Other mimics include bullous pemphigoid, pemphigus vulgaris, erythema multiforme, dermatitis herpetiformis, and epidermolysis bullosa acquisita (EBA). In children, chronic bullous disease of childhood (CBDC), also known as linear IgA disease, can resemble BLP [7].

Diagnosing BLP can be challenging due to its atypical presentation and resemblance to several other blistering disorders. A thorough clinical history and examination, along with laboratory investigations, histopathology, and direct immunofluorescence, are essential to rule out other immunobullous conditions. The hallmark histopathological features of BLP include hyperkeratosis, hypergranulosis, acanthosis, and the characteristic saw-toothed rete ridges. Additionally, the presence of large Max-Joseph spaces disrupts the dermoepidermal junction, leading to liquefactive degeneration of basal keratinocytes in both clinically involved and uninvolved skin [7]. This process ultimately results in the formation of tense subepidermal blisters typical of BLP.

Due to the limited number of cases and a lack of comprehensive studies, there are currently no established treatment guidelines for BLP. High-potency topical corticosteroids are considered the first-line therapy, while systemic corticosteroids are commonly used as second-line agents [6]. Some studies have also reported favorable responses to alternative therapies such as oral antimalarials and retinoids in managing BLP [6]. Although biologic agents are more commonly used for treating classical oral and cutaneous lichen planus, Alhubayshi et al. have demonstrated the successful use of adalimumab, a tumor necrosis factor (TNF) inhibitor, in achieving complete disease clearance in a case of BLP [6].

Our case adds to the limited literature by documenting a rare presentation of BLP associated with nephrotic syndrome, an association that, to our knowledge, has not been previously reported.

## Conclusions

This case makes a significant and noteworthy contribution to existing literature, as it is among the first documented instances of BLP occurring in association with nephrotic syndrome and severe renal failure. The rarity of this presentation, particularly in a young male patient, underscores the importance of maintaining a high index of suspicion for BLP in individuals presenting with atypical bullous eruptions, especially when accompanied by systemic conditions such as renal impairment. It highlights the need for careful clinicopathological correlation, including histopathology and immunofluorescence, to establish an accurate diagnosis and avoid misclassification with other bullous dermatoses.
